# Unraveling the Enigma of Cannabinoid Hyperemesis Syndrome: A Narrative Review of Diagnosis and Management

**DOI:** 10.7759/cureus.90961

**Published:** 2025-08-25

**Authors:** Shemyia A Smith, Mayar A Safwat, Brian J Piper, Maame A Addison

**Affiliations:** 1 Medical Education, Geisinger College of Health Sciences, Scranton, USA; 2 Center for Pharmacy Innovation and Outcomes, Geisinger College of Health Sciences, Danville, USA

**Keywords:** cannabis marijuana, droperidol, emergency medicine, haloperidol, ondansetron, topical capsaicin

## Abstract

Cannabinoid hyperemesis syndrome (CHS) is a serious condition primarily seen in chronic cannabis users, characterized by persistent cycles of severe nausea and vomiting, often leading to frequent emergency room visits. Accurate diagnosis is crucial due to the overlap with other gastrointestinal disorders. The traditional use of antiemetics such as ondansetron often fails to alleviate symptoms, making CHS difficult to manage. More effective treatments, such as haloperidol and topical capsaicin, have shown promise in reducing symptoms. Comprehensive management, including cannabis cessation, is essential. This narrative review explores current and emerging treatments for CHS, emphasizing the need for tailored therapeutic strategies and further research to improve patient outcomes. Pressing research needs include being able to predict who will develop CHS and optimizing interventions to reach those who are resistant to stopping cannabis use. As a narrative review, this study does not include statistical summary methods but emphasizes current knowledge and gaps to inform future studies.

## Introduction and background

Marijuana (*Cannabis sativa*) is a plant that is used all over the world. Cannabis is found in the Americas, Africa, Asia, Europe, and Oceania [1]. According to the 2021 World Drug Report, over 200 million people have used cannabis [1]. Due to the legalization of cannabis in some areas, there have been changes in the use and associated disorders across the United States. There has been an increase in the use of cannabis and a decrease in the prevalence of perceiving harm from smoking marijuana regularly [2]. Because many states have legalized marijuana, there has been an increase in the frequency of cannabis use in different locations. From 1992 to 2022, the frequency of cannabis users increased from 0.9 million to 17.7 million [3]. There were subtle differences in the amount of cannabis use in urban and rural areas. Depending on how one defines rural and urban, in the 2019 Ontario Student Drug Use and Health Survey, rural areas were known to drive sooner after their use and reported little to no consequences overall [4]. There was no significant difference (p = 0.350) in age and gender on the amount of cannabis use [4]. Besides age and gender, cannabis use disorders (CUDs) by race/ethnicity in the United States are increasing. All non-White groups had a higher percentage of cannabis use over time, with the majority being young adults (>16-35 years of age) [5,6]. Due to the liberalization of cannabis policies, there has been a renewed effort by biomedical investigators to understand the short and long-term effects of cannabis use. Given the variety of study designs and limited randomized clinical trials, we conducted a narrative review rather than a systematic review of the diagnosis and management of cannabis use.

In a recent report, almost half (47.9%) of cannabinoid hyperemesis syndrome (CHS) patients used cannabis daily, one-quarter (23.7%) more than daily, one-fifth (19.4%) weekly, and the rest (2.4%) less than weekly [7]. One of the main ingredients in marijuana is delta-9-tetrahydrocannabinol (Δ9-THC) [1,6]. After consuming high levels of Δ9-THC, some patients report to the emergency room (ER) for mild-to-severe conditions. In the ER, patients can be drug tested for cannabis in a few ways. Some ways patients can be tested are by their hair, urine, or blood. A urine test is the most common, quick, and efficient method. The detection time of immunoassays in a chronic heavy user is >30 days [8]. In whole blood samples, Δ8-THC and Δ9-THC can be detected. The detection window in blood is only a few hours, while hair has a much longer period [9]. High levels of THC have resulted in more short and long-term adverse effects, pathophysiological effects, and cannabis withdrawal symptoms [6].

Consuming too much cannabis over time can lead to CHS. CHS is a condition that affects millions of people throughout the world [10]. CHS can cause people to have cyclic episodes of severe nausea, abdominal pain, and vomiting [10-12]. Hyperemesis refers to prolonged vomiting [11]. There are three phases of CHS, i.e., prodromal, hyperemesis, and recovery. Each phase consists of symptoms associated with CHS, with symptoms worsening at each stage [11]. CHS could be due to a number of risk factors. These include where someone resides (i.e., local marijuana policies), the amount of cannabis use, potency, and health conditions. A patient history is necessary in diagnosing CHS. To be diagnosed with CHS, there must be a long-term use of cannabinoids (CBs) (natural or synthetic). It is important to take note of other conditions that may be misdiagnosed for CHS, such as cyclic vomiting or psychogenic vomiting [10]. There has been some speculation as to what causes CHS, but a direct cause has not been identified [12]. Preclinical (i.e., animal studies) research has determined that the CB dose is crucial, with low dose (5 mg/kg) cannabidiol reducing cisplatin-induced vomiting, but high dose (40 mg/kg) potentiating vomiting [13].

Cannabis can impact neurological and gastrointestinal organ systems [14]. Studies suggest that CHS can possibly be caused by overstimulation of several endocannabinoid receptors, such as CB type one and two (CB_1_, CB_2_) in the central nervous system (CNS) [7,15-18]. There are potential substrates that trigger the emesis center (EC) in the area postrema such as the cortex and limbic system (which controls stress, emotion, memory, smell, and taste), vestibular system (which controls balance), gastrointestinal (GI) tract (which responds to toxins, inflammation, obstruction, and stasis in the gut), and medulla (which controls vital process) [16]. THC stimulates these receptors, which causes nausea and vomiting, intestinal secretions, motility, and development of visceral pain [15,16]. Repeated exposure to CBs results in tolerance, which involves the desensitization of CB_1_ [19]. A transient receptor potential vanilloid (TRPV_1_) in the peripheral nervous system that is believed to trigger symptoms of CHS influenced by CB [20]. TRPV_1_ has a widespread distribution and has been identified in the dorsal root ganglia, trigeminal ganglion, as well as several areas in the CNS [21]. CB_1_ affects appetite, cognition, food control, and addiction [17]. CB_2_ primarily affects pain and inflammation [17]. The exogenous CB drugs often compete with the endogenous ligands for that receptor, which also affects brain maturation. For example, endogenous CB prunes synapses by interacting with receptors that regulate the release of glutamate and gamma-aminobutyric acid (GABA), while exogenous CB competes with receptors to inhibit pruning in specific brain areas such as the cerebellum [18]. Due to the receptors (CB_1_, CB_2_, and TRPV_1_) acting on the brainstem, CB triggers nausea and vomiting [22]. In the GI tract, endocannabinoids reduce gut motility and intestinal secretion through CB receptors [22].

CHS can be challenging to manage due to the limited effective treatments and the relapsing-remitting nature of drug addiction. Researchers have compared different types of drugs to help with the symptoms. Many of the treatments have been used for acute pain. Future studies and more research will need to be conducted for long-term care/cure. Although there have been earlier CHS reviews [7,14,23,24], the purpose of this narrative review is to describe the history and epidemiology of CHS and assess various treatments for CHS.

## Review

Methodology

A narrative literature review was conducted using PubMed and Google Scholar. The search terms used in each database were “Cannabinoid Hyperemesis Syndrome and case,” “CHS drug therapy,” “Cannabinoid Hyperemesis management,” and “Cannabinoid Hyperemesis management treatment.” No date range or journal exclusions were applied. Studies of all evidence levels and designs were evaluated for inclusion. Titles and abstracts were examined to establish their eligibility, and any discrepancies were discussed and resolved by the study investigators before data extraction.

Results

Diagnosis

**Figure 1:**
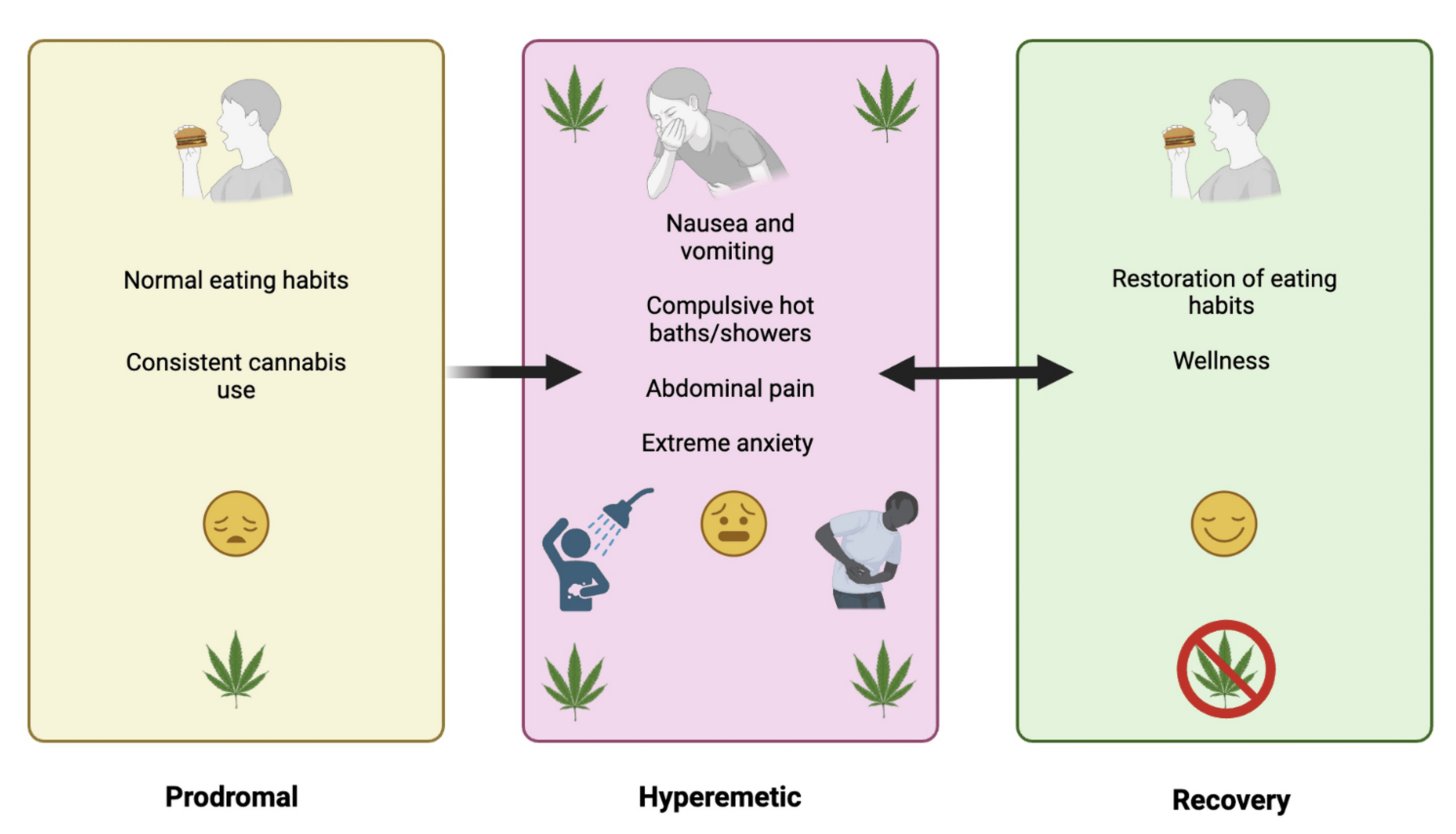
Phases of cannabinoid hyperemesis syndrome with associated clinical findings. Original image created by the authors using data from sources [23,27].

The diagnosis of CHS can be rather challenging due to its similarity in symptoms with alternative GI diseases. The foundational work of Allen et al. discovered that: (1) chronic marijuana use, often for several years, occurred before cyclic vomiting syndrome (CVS) symptoms, (2) a positive urine drug screen was identified in 10/10 cases, and (3) compulsive bathing was identified in 9/10 cases [20]. Denial of cannabis use is the biggest impediment to a CHS diagnosis [23]. A 98-person case series took issue with the requirement for an extended duration of cannabis use, as they discovered that 32.0% of patients reported using cannabis for less than one year before symptoms started [25]. The 2016 Rome IV CHS criteria mandates that for three months prior with symptom onset at least six months ago there is: (A) an episodic pattern of vomiting episodes, with the episodes lasting less than one week and asymptomatic periods greater than one week between episodes; (B) prolonged cannabis use; and (C) evidence of symptom relief by cessation of cannabis [26]. Criterion C may be difficult to meet in an ER setting if patients are only seen on a single occasion. Although criterion C is crucial for differentiating CHS and CVS, the majority of CHS patients may also have CUD. It may be challenging for those with CUD to stop cannabis use. The extent to which criterion C results in an underestimation of CHS cases is unknown. As shown in Figure 1, there are three phases in syndrome progression, namely, prodromal, hyperemetic, and recovery [23,27]. In the prodromal phase, onset occurs after cannabis consumption [23,27]. The prodromal phase involves early morning nausea, the development of a fear of vomiting, as well as abdominal discomfort. Patients recognize that cannabis appears to cause transient nausea relief. As consistent cannabis use progresses, the hyperemetic phase arises and symptoms such as nausea and vomiting, compulsive hot baths/showers, abdominal pain, and extreme anxiety manifest [23,27]. The hyperemetic phase involves intense and often incapacitating periods of nausea and vomiting as well as abdominal discomfort. Cannabis use continues or increases during this phase. Among patients who experience CHS, they very frequently engage in compulsive bathing behavior, which can become obsessive during the hyperemetic phase. The recovery phase involves cannabis cessation, which results in the reestablishment of normal eating habits and relative wellness [23,27] (Figure 1). One intervention that produced symptomatic relief was temperature control [20]. With a proper treatment plan, wellness can be restored once again [23,27].

Over half of CVS patients (n = 140) used hot showers or hot water bathing to alleviate symptoms [28]. Over two-fifths (41%) of CVS patients reported using cannabis in the past six months, and an appreciable subset (8.5%) met the criteria for CUD [28]. The key characteristic that differentiated CVS and CHS is that only CHS reported a complete resolution of symptoms with cannabis cessation [28]. Examination of diagnostic criteria for CHS in the adolescent population revealed that 100% of patients experienced recurrent vomiting, 99.4% had severe nausea with vomiting, 99.0% had abdominal pain, but only 23.0% had compulsive bathing habits [27]. These criteria serve as a challenge due to the overlap in symptoms, although it is notable that there was 100% reported experiencing cyclic vomiting. Similar to adults [28], symptom resolution was 100% with the complete cessation of cannabis [27]. Due to the stigma surrounding cannabis, some patients expressed concerns about whether their symptoms were thoroughly assessed by providers before a CHS diagnosis [29]. There is no International Classification of Diseases (ICD) code specific to CHS, although F12.188 has been used in ICD-10, which complicates longitudinal epidemiological research [30]. The code F12.188 is billed as cannabis abuse mood disorder with other cannabis-induced disorders [30].

The Visual Analog Scale (VAS) can be a beneficial tool for measuring the severity of these symptoms [31]. The symptoms of CHS can be assessed by the effectiveness of interventions in a hospital setting. The correlation between VAS and the Numeric Rating Scale (NRS) for scaling nausea severity among emergency department (ED) patients was determined [31]. The results showed a high correlation between both NRS and VAS, which identified the severity of nausea on a scale from none, mild, moderate, to severe [31]. When symptom relief was underway, this scale was a valid reflection of how the patient was feeling, thus providing further insights into diagnostic procedures.

**Figure 2:**
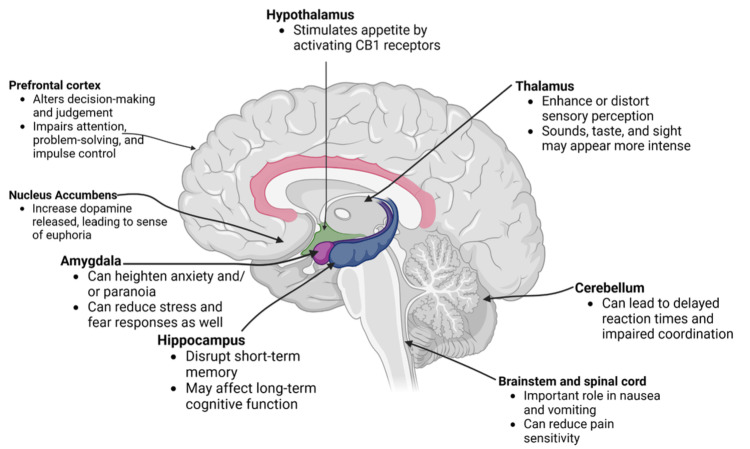
Effects of THC on different regions of the brain. Original image created by authors using data from sources [18,32,34-42]. THC: tetrahydrocannabinol

THC influences multiple brain areas, altering motor skills, cognitive functions, and emotions. In the hippocampus, it disrupts short-term memory, while in the prefrontal cortex, it impairs decision-making, judgment, impulse control, attention, and problem-solving [32,33]. The cerebellum is affected by delayed reaction times and impaired coordination [34], whereas the amygdala is associated with increased anxiety and paranoia [35]. The thalamus experiences distortions in sensory perceptions [36,37], and the nucleus accumbens shows increased dopamine release, leading to a sense of euphoria [38]. Figure 2 displays the brain regions that are acutely affected by THC [18,32,34-42].

Epidemiology of Cannabinoid Hyperemesis Syndrome

Marijuana use has undergone substantial changes in use over time. According to the nationally representative Monitoring the Future, over half of high school seniors had used marijuana in the past 12 months in the 1970s, but use patterns are now considerably lower, although still common. Over one-eighth (13.0%) of high school seniors used marijuana daily in the past month in 2024 [43]. There were 43.6 million people aged 12 or older in the United States who used marijuana in the past month [44]. In 2019, there were 25 million people aged 15 or older in the European Union who used marijuana in the past year [1].

CHS has had an increase in prevalence among regular cannabis users [45]. The estimated prevalence of CHS varies across different populations [45]. First described in Australia in 2004 [20], it is a generally under-recognized entity that affects heavy, chronic marijuana users [45]. In one retrospective study, there were a total of 155 patients in the ED who identified as daily cannabis users, and 54.2% were between the ages of 18-29 years [45]. Along with the estimated 8.364 million daily marijuana smokers in the United States in 2014, there were about 2,130,000 to 3,380,000 individuals who suffered from CHS symptoms [45]. Almost one-third of the sample met the criteria of CHS and reported hot showers as a relief method [45]. Analysis of the US nationally representative Nationwide Emergency Room Sample revealed that suspected CHS-related ER visits among individuals aged 15-24 years increased 25-fold from 2006 (6.8 cases per million population to 2020 (173.2) [46]. One possibility is that the increased visibility of this topic resulted in fewer missed diagnoses. If THC increases and CBD inhibits CHS symptoms (as might be predicted from the preclinical research) [13], it may not be a coincidence that an analysis of seized samples determined that the THC to CBD ratio increased four-fold from 24.8 in 2009 to 103.5 in 2017 [47].

These symptoms of CHS lead to multiple ER visits, as shown via a six-year audit of adult presentations to an urban district hospital [48]. The key findings included the delays in diagnosis. The research of 142 adults, most being young males with frequent cannabis use, identified elevated white cell counts and mild hypokalemia [49]. Having this as an additional marker for the indication of CHS could be beneficial in targeting early diagnosis and developing improved treatment options. The mitigation of early diagnosis can contribute to the improvement of healthcare costs [50]. Patients suffering from CHS have considerably greater health costs, and obtaining proper social history from patients in the hopes of early detection is desired [50]. Financial relief from diagnostic procedures that can be medically unnecessary can improve these rather comprehensive evaluations [50].

Treatment

Management of CHS requires judicious diagnosis to treat the presented symptoms. Haloperidol has been demonstrated to be a superior choice for emergency treatment of nausea, including the treatment of CHS in the ER [51]. Haloperidol functions through dopamine D_2_ receptor antagonism, which has a complex relationship with CB receptors, shown through studies examining the relationship of the two receptors with Δ9-THC [52,53]. Droperidol, a butyrophenone and D_2_ antagonist, is an effective sedative, anxiolytic, analgesic, and antiemetic medication [54]. Intravenous sroperidol, if given too rapidly, may produce adverse effects of apprehension and anxiety [55-57]. Haloperidol can also be used off-label to treat anxiety, which may be beneficial for understanding why patients treated with haloperidol had fewer hospital readmissions [58]. Concerns with haloperidol include neuroleptic malignant syndrome and potentially prolonging the QT interval [59]. Benzodiazepines are also effective for CHS, especially in conjunction with agents in the butyrophenone class [60]. The prevalence of treatment for cannabis users was about 6 per 1,000 in young people [61].

Droperidol has a more potent and rapid onset than both ondansetron and haloperidol, given its low dosage and primary use as an antiemetic, sedative, and antipsychotic [54-56]. Ondansetron did not fare as well compared to droperidol, with subjects requiring more rescue medication and pain management [55]. The relationship of these two drugs with CHS sheds light on the poorly understood mechanism of CHS. Ondansetron is a 5-hydroxytryptamine 3 (5-HT_3_) receptor antagonist and works mostly with chemoreceptors. CHS is believed to be triggered by overstimulation of the CB receptor, which does not interact with the 5-HT_3_. For this reason, ondansetron and other antiemetics in the same class do not have much effect on those suffering from CHS [62]. Acute management typically involves supportive therapy with intravenous fluids and antiemetics, followed by correction of electrolyte imbalances resulting from emesis [7]. Alternative treatment options have also been explored, such as topical capsaicin, which has been the emerging CHS treatment [63]. Topical capsaicin reduced nausea and vomiting in both pediatric and adult patients after direct application to the abdomen [63]. This provided a non-invasive treatment as opposed to the ingestion of traditional antiemetic drugs [63]. Capsaicin cream has benefits in treating CHS, using the TRPV_1_ receptor as its mechanism of action, which is involved in the regulation of pain and nausea [64]. Other advantages include low cost and over-the-counter availability. On the other hand, the prescription formulation (8.0%), which was approved for neuropathic pain in 2009, has not been evaluated for off-label CHS use [24].

These administered drugs, although effective, may only provide acute relief from symptoms [65]. A prospective investigation that followed patients after visiting the ER identified high symptomology in the following two weeks, and the vast majority (87.0%) did not stop cannabis use in the next three months [66]. Overall, the most effective and only cure for CHS is complete abstinence from cannabis [65]. The combination of support from primary care providers and therapists may be a valuable tool in patient support and can prevent recurrence [65]. As patients may be unaware of these symptoms and their connection to marijuana use [29], it is rather crucial that, with professional support, medically and psychologically, and early symptom management, cannabis abstinence can be achieved [65].

**Figure 3:**
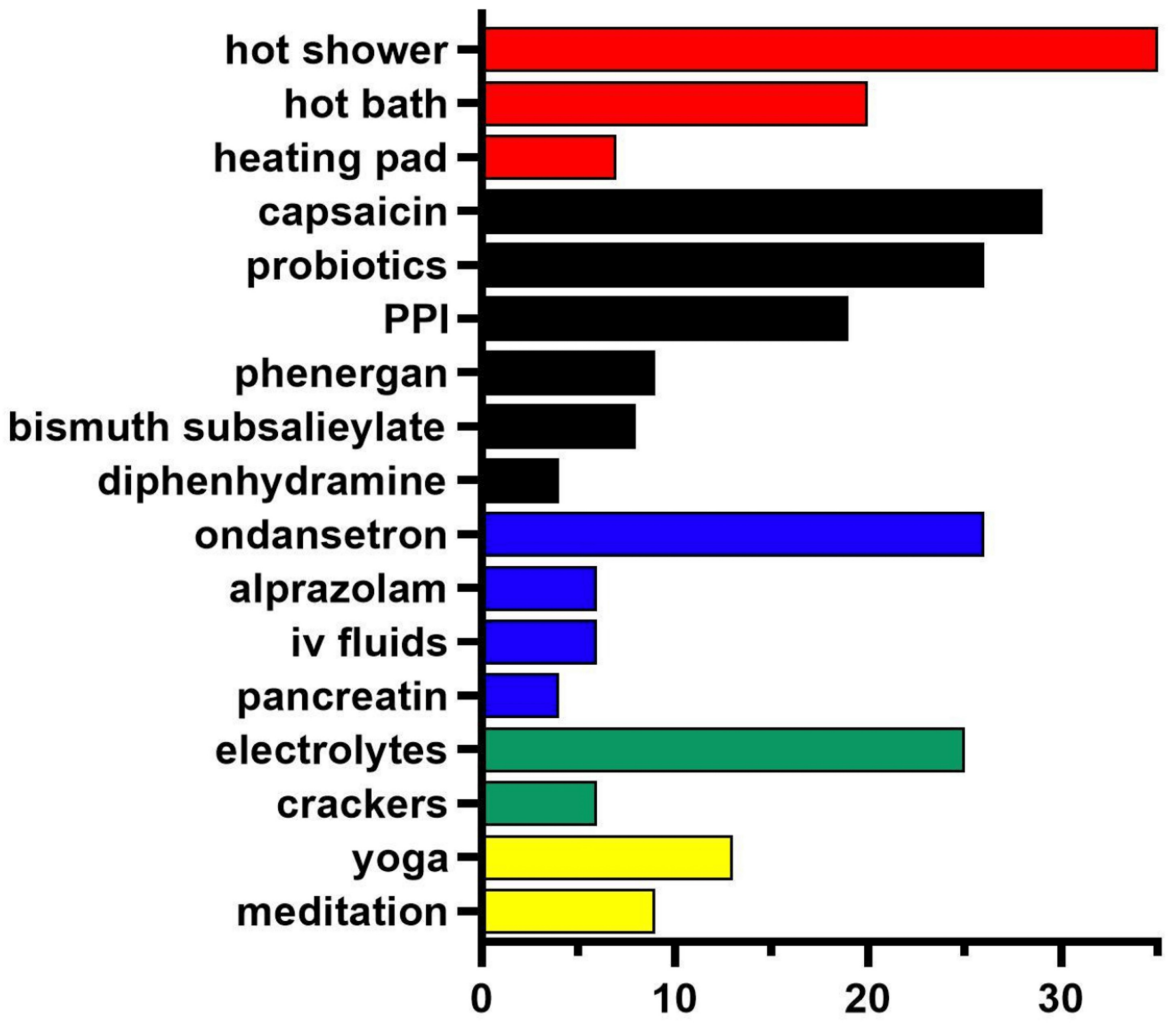
Treatments and home remedies used for cannabis hyperemesis syndrome, including manipulating temperature (red), over the counter drugs (black), medicines (blue), foods (green), and alternative medicines (yellow), based on Reddit posts (N = 69) with at least 30 upvotes. Original image created by the authors using data from source [68]. PPI: proton pump inhibitors

There is no shortage of remedies employed for CHS. A particularly innovative report leveraged social media to examine Reddit posts from 2018 to 2022 from six subreddits (e.g., r/CHSinfo). Figure 3 shows the findings of the 69 posts that each had at least 30 upvotes. The most common therapies that were posted about included hot showers, capsaicin, probiotics, ondansetron, and electrolyte-containing drinks [66].

**Figure 4:**
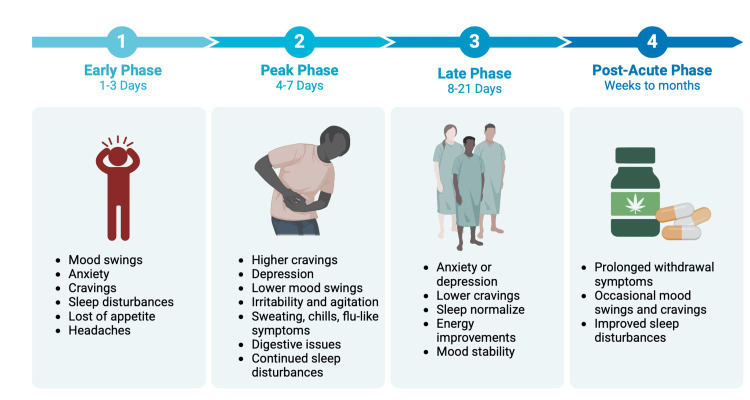
The four different phases of CWS. Original image created by the authors using data from source [68]. CWS: cannabis withdrawal syndrome

Cannabis withdrawal syndrome (CWS) presents an additional confounding factor that can pose a challenge in management. CWS can trigger patients into a relapse of cannabis use and serve as a psychological reinforcement [67]. As shown in Figure 4, there are four phases of CWS, namely, early, peak, late, and post-acute [68]. Each phase affects the limbic system, and the symptoms improve in each phase over time.

Complications once again arise when this diagnosis is not promptly managed. Recurrent vomiting can lead to electrolyte imbalances and renal failure [69]. CHS exists in palliative care when cannabis is used as a treatment modality when there are no other disease-modifying treatment options [69]. To mitigate this, treatment protocols should be developed to create a standard. This can be done with the analysis of long-term outcomes and via the integration of not just medical interventions but behavioral interventions as well [70].

**Figure 5:**
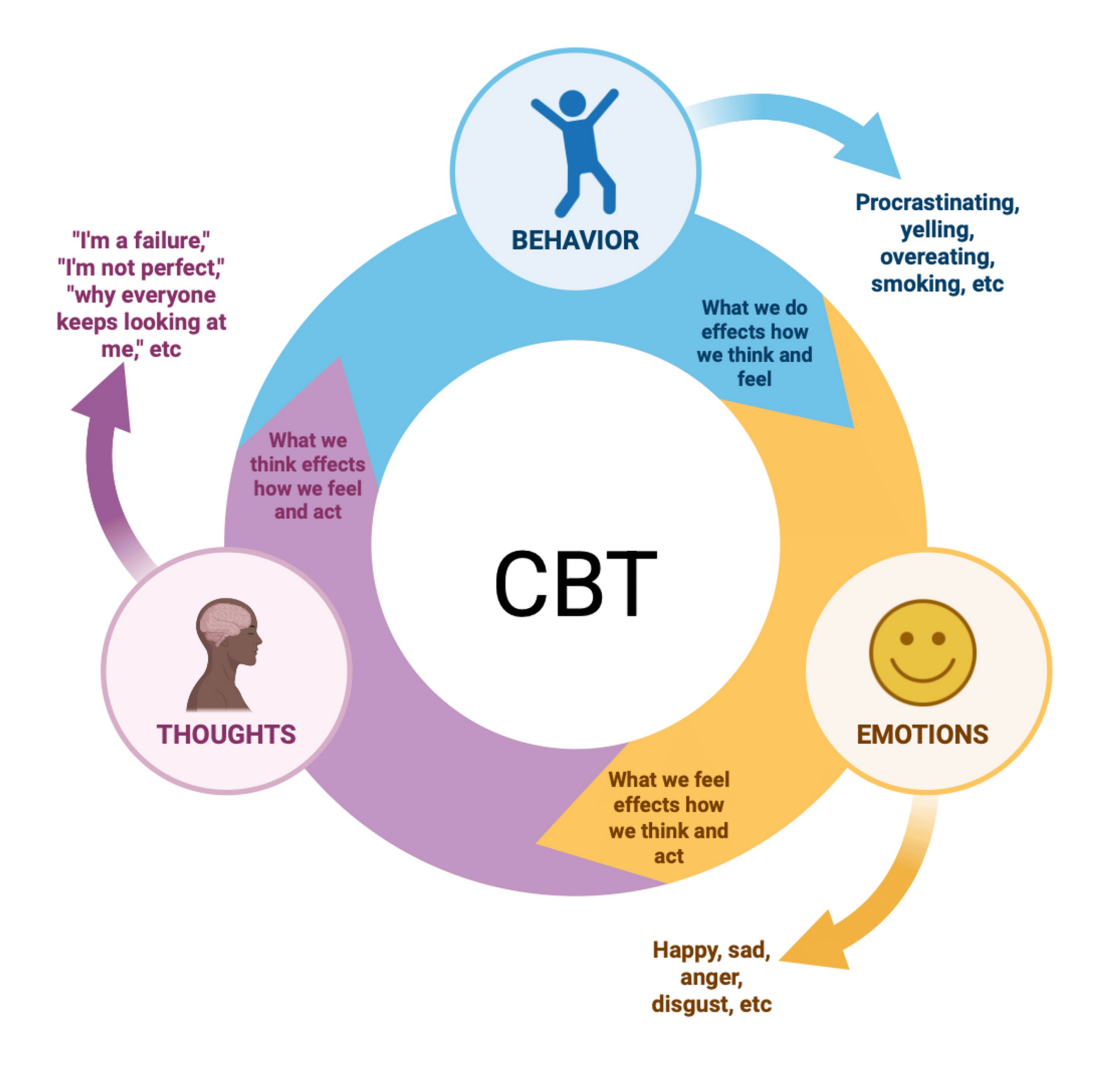
Overview of CBT. Original image created by the authors using data from authors and source [71]. CBT: cognitive behavioral therapy

As cannabis has grown be a considerable concern, the treatment for those who suffer from CWS has become challenging. Due to the excessive use of cannabis, the endocannabinoid system may dysfunction [71]. In terms of CWS, it is easier for some patients to reduce the amount of cannabis over time. A study was conducted to show the clinical and functional outcomes of those who ceased and/or reduced cannabis use versus those who did not stop [71]. After a 12-month follow-up visit, results indicated that there was a decrease in general psychotic symptoms in those who chose to stop and or reduce the use of cannabis (β = -0.754; p = 0.0222 to β = 0.197; p = 0.050) [71]. There are several techniques used to help with CWS, such as healthy lifestyle changes and cognitive behavioral therapy (CBT). Informing patients of the consequences of using cannabis and what it can do to their bodies is the first step. One way researchers have found to help reduce the symptoms is CBT [71]. CBT is known to improve physiological functions and reduce the use of cannabis. Behavioral therapy is known to help manage anxiety, stimulus control, and identify triggers, as shown in Figure 5 [71]. Cognitive therapy is known to help by introducing coping skills, cognitive reconstruction, and assertiveness [71]. One of the most efficient ways to reduce cannabis use is by switching to a healthier lifestyle. However, exercising and dieting can be cost-efficient, accessible, and low-stigma [72]. Exercise can be a good choice because it can activate the production and release of endocannabinoids and endorphins [72]. Exercise has shown improvements in physiological and behavioral responses, without having excessive adverse effects. Overall, exercise is a step in helping restore the endocannabinoid system in CUD patients and can be used as a form of therapy. 

The severity of CWS varies with the amount of cannabis ingested [73]. Therefore, the physical symptoms of CWS, such as nausea and stomach upset, fluctuate [73]. Gabapentin, when given at a dose of 1,200 mg/day, showed approximately a 30.0% improvement during the 12-week treatment plan [74]. The neuro-enhancing effects of this medication aid the hypothalamus in regulating mood and sleep, which are key factors as to why many cannabis users relapse [74]. Chronic use of cannabis can lead to an overactivation of corticotropin-releasing factor (CRF) in the amygdala [74]. Gabapentin stabilizes CRF-induced GABA activation within the amygdala [74]. The calcium channel GABAergic mechanism aids in the brain’s homeostatic systems, especially in the prefrontal cortex [74]. In a study of gabapentin versus placebo, gabapentin drastically reduced the resumption of marijuana [74]. This study consisted of a randomized trial with each subject meeting the baseline criteria of the Diagnostic and Statistical Manual of Mental Disorders, Fourth Edition [74]. Overall, each person was observed for several weeks with a checklist, including psychological and physiological manifestations of marijuana withdrawal monitored for severity each week on a zero to three scale, zero being not severe and three being severe [74]. The participants of this assessment reported a mean severity rating of about one and a half, which corresponds to mild-to-moderate severity [74]. This serves as a great alternative management option, as this pharmacological method was tolerated by most subjects and had promising results in the route to complete cessation [74].

Pathophysiology

Similar to CVS, the physiological defects responsible for CHS are incompletely understood [26]. Marijuana has been utilized for medical and recreational purposes for at least 2,500 years [75], but modern medicine continues to learn about the adverse effects it produces. We focused on one of those potential adverse effects, CHS, which is somewhat paradoxical as marijuana has been used to mitigate nausea and vomiting [76,77]. As noted earlier, CHS was defined as a disease in 2004, yet the diagnosis of CHS has only recently started to increase [78]. CHS is presumed to be caused by the overstimulation of an endocannabinoid receptor, causing nausea and vomiting [17]. CB_1_ is expressed most densely in the cerebral cortex, amygdala, basal ganglia, hippocampus, and cerebellum, and at moderately high levels in structures associated with pain, such as the dorsal root ganglia, periaqueductal gray, rostro-ventral medulla, and the limbic system. CB_1_ is also found in non-neuronal tissue, including the liver, adipose tissue, and pancreas. CB_2_ is expressed most densely in immune tissues. Although brain levels of CB_2_ are lower, this receptor is still important for pain. In addition to CB_1_ and CB_2_, there are also orphan G-protein coupled receptors (GPRs) (GPR-3, GPR-5, GPR-12, GPR-18, and GPR-55) that may also be important in the response to CBs. However, neuroadaptations in the response to repeated exposure to CBs are thought to be due to changes in CB_1_ [19]. Cannabis has been used as a therapeutic treatment for pain and emesis [79,80]. Receptors of cannabis can act along the neurological and GI organ systems. This could mediate vomiting, which is ironic that a drug used as an antiemetic can cause vomiting, and even CVS could cause such distressing episodes of vomiting [81,82]. Studies have identified CBs, such as THC, CBD, and tetrahydrocannabinolic acid, when administered a single time, can limit nausea and vomiting [83-85], but less is known about their efficacy with repeated administration. It may not be a coincidence that the concentration of CBD, which can inhibit vomiting at low doses [13], in seized samples of illicit cannabis products in the United States decreased from 0.5% in 2004 to <0.2% in 2014 [86].

The cannabis plant is a bioaccumulator that absorbs heavy metals from the soil [87]. Among 16 German patients who were admitted to the hospital following consuming lead-adulterated marijuana (presumably to increase the weight and therefore profit), 16 (100.0%) had nausea, vomiting, weight loss, and fatigue, 15 (93.4%) had abdominal cramps, and eight (50.0%) had Burton’s line [88]. It might be informative to conduct analytical chemistry for heavy metals on the cannabis products used by CHS patients.

Hot water hydrotherapy is a mainstay of self-management of CHS [66]. Regions of the hypothalamus have distinct roles in thermoregulation, with the anterior being responsible for heat dissipation and the posterior for conserving heat [89]. In experimental animals (mice and rats), CB_1_ partial or full agonists cause hypothermia. However, there is tolerance to this effect, which suggests neuroadaptations, at least in experimental animals [19]. Although exposure to elevated water temperatures is commonplace, it may be easy to overlook the profound physiological effects that this induces [90], as well as a sensation of relaxation and decreased anxiety. Immersion in 39℃ for over 20 minutes by health volunteers produced the anticipated elevation in core temperature (+0.5℃) but also increased heart rate (+21.6 beats per minute), as well as decreased systolic (-11.6 mmHg) and diastolic (-25.8 mmHg) blood pressure [91]. Further study is necessary to identify the neural substrates for nausea and thermoregulation that are responsible for the transition from irregular recreational use to the prodromal phase and from the prodromal to the hyperemesis phase.

Research to identify the genomic risk factors for CHS is limited to one report. In a study involving 99 patients with diagnosed CHS who agreed to participate, only 28, as well as 12 CHS-negative controls, returned the genetic kit [92]. Despite the modest sample, the study identified mutations in several genes, including *COMT* (odds ratio (OR) = 12.0), *CYP2C9* (OR = 7.8), *TRPV_1_* (OR = 5.8), and *CYP2C19* (OR = 4.6) [92]. Being able to better understand why only a subset of the many cannabis users will develop CHS is urgently needed.

Case reports

The CHS literature is replete with case reports and case series [20,25,93]. Although case reports are not informative for causality, they may be valuable for hypothesis generation [94]. A 1996 case of a 22-year-old man attributed psychogenic vomiting, which was relieved by hot showers, to maternal separation anxiety disorder (SAD) and not to his eight-year history of smoking marijuana [95]. This field was originally more focused on recreational use [20], although an increasing number of reports are of medical use [96]. Many reports are of younger adults, predominantly males [20,25,97]. There are many reports in pregnancy [98,99] and in pediatrics (Table 1). Reports in the elderly are currently uncommon [65,70,100]. Age <50 years at evaluation has been proposed as a supportive criterion [25]. However, the Rome IV diagnostic criteria do not list this [26]. It is possible that there will be more older adults identified with CHS in the future. An important caveat in interpreting the case literature is that Criterion C is often not feasible in acute care settings. A systematic review of cases and case series published from 2000 to 2018 determined that only 16.2% (44/271) had a follow-up >4 weeks. Therefore, only about one-sixth of the cases reported as CHS fulfilled all the Rome IV criteria [101].

**Table 1:** Pediatric cases of cannabinoid hyperemesis syndrome.

Study	Age	Sex	Key information
Lonsdale et al. [160]	13–20	Males and females	21 of 34 (62%) cases were females
Miller et al. [152]	17	Male	Heavy, daily marijuana use since age 14. Symptoms resolved 1 month after cessation
Miller et al. [152]	18	Female	Marijuana use since age 16, including 4-5 blunts/day. Taking up to 7 showers/day
Klazura et al. [159]	17	Female	Daily smoking since age 15. Pneumomediastinum managed medically.
Wilson [158]	17	Female	27.2 kg (60 lb) unintentional weight loss over 4 months
Desjardins et al. [146]	17	Male	Nausea not relieved by intravenous ondansetron, dimenhydrinate, and metoclopramide
Merino et al. [151]	15	Female	Unintentional 22.7 kg weight loss in the prior 6 months. Haloperidol infusion stopped when a long QTc (528 ms) was identified
Graham et al. [147]	16	Female	Following application of capsaicin cream (0.025%), pain reduced from 6 to 3 out of 10
Brown et al. [60]	16–18	Males and females	Haloperidol, lorazepam, and/or topical capsaicin showed symptomatic relief for six adolescents
Patterson et al. [124]	18	Female	Outpatient haloperidol (5 mg) produced a complete symptom resolution in a patient who used cannabis 2-3 times/day but was unwilling to discontinue cannabis
Cordova et al. [144]	16–22	Males and females	Quantitative urine analysis of THC-COOH >100 ng/mL is an index of chronic cannabis use

After Allen et al.’s seminal case series of nine patients with a two-year follow-up, which was published in 2004 [20], another report from Australia documented the extent to which CHS and compulsive bathing could impact a patient’s life. A 30-year-old man who had used up to 3 g/day of marijuana was reported by his wife to spend all day in the bathtub 300 out of 365 days. He was only able to work one to two days per week due to his symptoms, and he would sometimes stay in a hotel because of the availability of unlimited hot water [102]. A 32-year-old man became agitated and aggressive when denied access to the ER showering facilities [103]. A case series with eight adult patients determined that the average duration of bathing was five hours per day [104]. In addition to the tremendous suffering and adverse impacts on multiple aspects of one’s life, the medical resources expended pre-CHS diagnosis may not be trivial [105]. A 22-year-old man had 13 ER visits and four abdominal-pelvic CT scans for nausea, vomiting, and abdominal pain over 25 months [106]. A 34-year-old man had a prophylactic cholecystectomy, which did not resolve his cyclic vomiting, before it was discovered that he had a nine-year history of daily marijuana use [107]. A 32-year-old man who presented with cyclic episodes of nausea and vomiting was hospitalized 24 times in a two-year period. While hospitalized, he was observed to take multiple hot showers, which relieved his symptoms [108]. An analysis of an unusually large case series (N = 48-98 depending on the measure) revealed that the CHS pain was variously described as crampy (29.0%), burning (27.0%), and sharp (23.0%). The time to symptom improvement after diagnosis was one to three months. Also notable was that 27.0% had a body mass index <20 kg/m^2^, which could be reflective of the protracted periods CHS patients may go before diagnosis [25].

A few findings from the French addictovigilance network’s 29 cases are noteworthy. They found that both antiemetics and dopamine antagonists, such as metoclopramide, metopimazine, and phloroglucinol, were not effective, although no information about the route of administration or dose was provided. This report distinguished between the effect paradox, that cannabis is often used for treating nausea but causes presumably dose-dependent nausea in CHS, and the temporal paradox, in which the first CHS case in France was only identified in 2013 despite an extensive history of prior use in France [109]. Traditional Chinese Medicine and the bencao texts from 200 AD onward recognized the medical applications of cannabis for epilepsy, seizures, pain, and later as an anesthetic and for treating severe spasms, as well as for causing “happiness in the heart.” These early texts also identified what are now considered the adverse effects of inducing a state of drunkenness [110]. The almost doubling in THC potency from French samples obtained in 2000 (9.0%) to 17.4% in 2013 is proposed to account for the temporal paradox [109].

There is a preponderance of case reports on botanical marijuana as the causative agent [20,25]. However, there are also instances implicating Δ8-THC, nabilone, the full CB_1_ agonists, and even cannabidiol [70,111-115] (Table 2). As there is some stigma associated with cannabis [116], asking patients “Have you tried marijuana for vomiting?” may be an easy way to approach this sensitive topic [117]. There are currently no reports where dronabinol was the causative agent, although it should be recognized that US use of this oral prescription medication is quite limited [118]. Dronabinol has been employed to aid in the discontinuation of smoked marijuana [119,120]. On the other hand, dronabinol has worsened CHS symptoms [121].

**Table 2:** Key case reports or case series in the literature on cannabis hyperemesis syndrome. ALS: amyotrophic lateral sclerosis; JWH: John W. Huffman; ME: medical examiner; THC: tetrahydrocannabinol

Study	Age	Sex	Agent	Specifics
de Moore GM et al. [95]	22	Male	-	Psychiatric causes presumed responsible for continuous vomiting
Allen et al. [20]	Varied	Males and females	Marijuana	Compulsive bathing in 9 of 10 patients including waking at night for showers
Chang et al. [135]	23	Male	Marijuana	Visited ER several times after his hot water supply (4 hours) was exhausted
Bonnet [121]	26	Female	Marijuana	BMI at 14.5 when diagnosed. Dronabinol worsened vomiting attacks.
von Both I et al. [143]	22	Female	Marijuana	Mutations in MYBPC3 and RYR2 identified in Torsades de Pointes induced lethality following administration of three QT prolonging agents (haloperidol, ondansetron, and olanzapine)
Howard [96]	31	Male	Cannabis oil	CHS symptoms resolved 3 weeks after the discontinuation of smoked cannabis oil. This ALS palliative care patient continued the use of whole plant-based edibles
Senderovich and Waicus [70]	70	Female	Nabilone	Discontinuation of nabilone resolved nausea and vomiting. On reinitiation, symptoms returned
Rosenthal et al. [111]	38	Female	Δ8-THC	Gummies were used most nights for sleep
Ukaigwe et al. [112]	38	Male	K2 and MJ	Cannabicyclohexanol was not detected in conventional urine drug screens
Hopkins and Gilchrist [113]	30	Male	JWH-018	Analytical chemistry identified JWH-018 and 073 in blood and urine, but was negative for THC
Katz et al. [114]	11	Male	Cannabidiol	Emesis resolved in 2 months after the prescription of cannabidiol (15 mg/kg/day) was discontinued
Lefebvre et al. [115]	24–32	Male	Cannabidiol	The patient had stopped using marijuana for 7 months, but was smoking cannabidiol
Laborde-Casterot et al. [125]	41	Female	Cannabidiol	Hyperemesis, relieved by hot baths, developed after two months of CBD use

There are half a dozen reports of patients that have tested whether cannabis cessation is an absolute requirement for an improvement in their CHS symptoms [7,96,101,106,122,123]. An ALS patient was able to end their CHS symptoms after stopping smoked cannabis oil but reported that they continued with edibles [96]. The edibles, with a self-reported mean THC of 10 mg, were used three to five days a week [96]. A 22-year-old man who smoked two to three joints per day slowed down on his marijuana use, following which his symptoms dissipated [106]. There are two minimally described cases of CHS symptomology developing after switching to a new CB product, which resolved after they returned to their original product [7]. A patient who met the Rome IV CHS criteria quit cannabis for at least one month and reported a complete resolution of symptoms. Further, this patient subsequently remained episode-free after resuming cannabis use with a greater CBD to THC ratio [101]. A 33-year-old woman, who was also prescribed an antipsychotic, olanzapine, to take as needed, found that decreasing the number of joints by 50.0% (i.e., from two to one per day) decreased the number of hot showers she required from eight or nine down to one per day which was only for hygiene purposes [122]. Similarly, a 36-year-old man, who was also prescribed an atypical antipsychotic, reduced his daily smoking by 75% (i.e., from four to one per day) and had no vomiting episodes during one year of follow-up [123]. These instances raise the possibility that hyperemesis is dose-dependent or there is a CB, typically thought to be THC, threshold that patients could stay below if they were unable or unwilling to achieve complete abstinence. On the other hand, it is also crucial to appreciate that, following abstinence, CHS symptoms typically return with the resumption of cannabis use [20,104,124].

There are three reports of CHS associated with cannabidiol [114,115,125]. A 41-year-old woman taking 10 to 30 drops of 50 mg/mL CBD oil at least three times per week for two months developed hyperemesis, in association with abdominal pain and nausea, which was alleviated with hot baths. An analysis of four 2 cm hair segments revealed a median CBD concentration of 290 pg/mg and a low (7 pg/mg) THC concentration [125].

Recurring dermal injuries from repeated exposure to very high temperatures have been identified [126-129]. A woman in her 50s with a 38-year history of smoking cannabis removed the cover of the heating pads to increase the temperature and apply this continuously. She developed a cutaneous disorder caused by chronic heat exposure, erythema ab igne [127]. The compulsive hot showers for a 36-year-old female with CHS resulted in repeated episodes of severe burns, sepsis, and hospitalizations. However, it was unclear whether cocaine’s anesthetic properties contributed to these adverse outcomes [128]. Notably, the American Academy of Dermatology (AAD) does not make a strong recommendation regarding optimal bathing frequency in adults [130]. However, for those in their 60s and 70s, the AAD recommends warm, not hot, water as hot water strips skin of its natural oils, which can cause dryness, and to limit bath or shower duration to 5-10 minutes [131].

There are several reports of CHS having indirect effects on kidney function [132-137]. A 36-year-old man presented to a psychiatric clinic. His laboratory values indicated acute renal failure and electrolyte abnormalities. His symptoms resolved within 48 hours of cannabis cessation [134]. A 28-year-old man with a history of smoking five joints/day since age 14 presented to the ER with tachycardia and orthostatic hypotension, suggestive of hypovolemia. His serum and urinary electrolyte status showed metabolic alkalosis, hemoconcentration, and extracellular dehydration. Following instructions to stop smoking cannabis, he was subsequently hospitalized for acute renal failure five times in a two-year period [133]. A 25-year-old man with long-term marijuana use, using 2 g to a quarter oz daily for the past eight years, presented to the ER for vomiting “at least twenty times a day.” He was spending about 50.0% of his awake time in the shower. His creatinine (3.21 mg/dL) and blood urea nitrogen (BUN) (24 mg/dL) were elevated. He was admitted for acute renal failure secondary to the dehydration induced by the vomiting, hot showers, and high ambient temperatures. The creatinine and BUN normalized after one day of intravenous hydration [136]. Although there is one case report that hypothesizes that the dehydration and electrolyte imbalances of CHS increase the risk for nephrolithiasis [94], other epidemiological research does not support this [138].

Although the CB_1_-mediated effects on the cardiovascular system occur through sympathetic activation and parasympathetic inhibition, a less studied putative CB receptor, GPR-55, may also be involved in cardiovascular homeostasis and disease [139]. There are a handful of CHS cases involving cardiac dysfunction [107,140-143]. CHS impacted the management of the coronary artery disease of a 36-year-old woman [144]. A 22-year-old woman died from her cardiac arrhythmia in combination with vomiting-induced hypokalemia and receipt of QT interval prolonging medications [143]. However, it should also be noted that there were no serious cardiac reports in a 98-patient case series, which suggests that cardiac events are uncommon [25].

There is moderate-sized pediatric literature [27,144-159] (Table 1). Despite the preponderance of reports on THC-containing products, an 11-year-old boy receiving cannabidiol (15 mg/kg/day) for refractory epilepsy developed severe bouts of emesis, each lasting 24 to 48 hours and separated by one month, at age 13. His emesis resolved two months after weaning off cannabidiol [114]. Interestingly, there were more females (62.0%) among pediatric cases in a large (N = 34) case series from the Johns Hopkins Children’s Hospital in Florida. The authors noted that 91.0% of their cases (31/34) did not meet all the adult diagnostic criteria for CHS. This was typically due to the “relief of vomiting episodes due to sustained cessation of cannabis use” Rome IV criteria [26,160], which suggests that adolescent-specific diagnosis may be needed. In a previously reported study, a presumably well-intentioned mother was illegally purchasing marijuana to treat her 17-year-old son’s nausea and vomiting. He was smoking four to five joints per day and had been to the ER two to three times per week for the past three weeks for nausea, vomiting, and epigastric abdominal pain. The staff were concerned that he was a drug seeker interested in the single dose of hydromorphone he received for abdominal pain [148].

A 2021 systematic review of pediatric CHS identified 10 reports focusing on diagnosis (57.0% female) and 11 reports focusing on treatment (64.0% female). Capsaicin cream and haloperidol were characterized as effective in some reports [27,147]. The adverse effects of haloperidol, including dystonia, extrapyramidal reactions, neuroleptic malignant symptom, and long QTc, make capsaicin particularly appealing for use with adolescents. A common adverse effect of topical capsaicin cream is a mild burning sensation on the abdomen [147].

Future case reports or series might benefit from obtaining more detailed information about which cannabis product(s) were involved. For example, one report of retching contributing to hypertensive crisis in a 69-year-old man with pheochromocytoma noted that he had a 1.5-year history of smoking an 88.0% THC smoke pen and taking five to six hits per day, and smoking cannabis joints two to three times per day [100]. There is non-human animal research indicating that low-dose cannabidiol can reduce vomiting [13]. It is not currently known if there is an optimal amount of CBD that could limit CHS development in humans. However, analysis of marijuana seized by law enforcement revealed that CBD potency has declined while THC potency has increased [47,86]. A handful of US states (Connecticut, Louisiana, New York, Ohio, and Virginia) include medical marijuana in their Prescription Drug Monitoring Program [161]. This can be a resource to complement the self-reported history and begin to characterize if high-potency and smoked administration is more likely to result in CHS. A quantitative urine analysis may be more informative than dichotomous THC screens because it measures the amount of concentration of substances within urine [144]. The case reports suggest that there is variability in receptivity to cannabis cessation [20,25]. It is currently unknown if tolerance breaks can decrease the likelihood of CHS development. Long-term prospective studies will be informative to better understand the outcomes and rates of relapse for this challenging condition [162]. It will also be important to conduct mechanistic studies with positron emission tomography and other neuroimaging methods to determine the neurobiological substrates (e.g., CB_1_, TRPV_1_, or D_2_ receptors, including in the area postrema) that distinguish between CUD and CHS. The ER is a valuable resource. The number one and number five causes for ER visits were abdominal pain, with an average cost per visit of $5,111, and mental health and substance abuse, with an average cost of $3,166 [163]. Efforts to maximize CHS outcomes, including cessation, while limiting ER visits, will be beneficial. As heavy metal ingestion can produce nausea, vomiting, and abdominal pain, analytical chemistry of blood, urine, and the cannabis products consumed for lead and cadmium may be informative [164]. There is also a need for head-to-head randomized controlled trials of an antipsychotic versus capsaicin, examining both efficacy and adverse effects in adults as well as pediatric samples.

## Conclusions

An objective of this narrative review was to form a foundation to more optimally diagnose and treat CHS in the ER and other settings. The protracted experience of some patients before CHS diagnosis is a continuing challenge. The origin of the effect paradox and the temporal paradox is currently ambiguous. As there are US FDA-approved cannabinoid drugs for nausea and vomiting, additional study is urgently needed to identify the pharmacogenomic or cannabis profile that increases or decreases the risk of CHS development. This includes determining if certain routes of administration or higher THC potency are risks for developing CHS. The absence of an ICD code specific to CHS until recently could result in under-reporting in epidemiological investigations. Across reviewed studies, haloperidol was reported as more effective than ondansetron. Lower doses of haloperidol were also effective and had a lower chance of causing adverse effects. Future studies should focus on understanding why haloperidol has been observed to be effective in reducing repeat ER visits. This could potentially be achieved by exploring the mechanistic aspects of haloperidol in relation to CHS to develop even more efficacious pharmacotherapies with fewer adverse effects that can be employed until cannabis cessation is achieved.
